# Identification of biomarkers differentiating Alzheimer’s disease from other neurodegenerative diseases by integrated bioinformatic analysis and machine-learning strategies

**DOI:** 10.3389/fnmol.2023.1152279

**Published:** 2023-05-10

**Authors:** Boru Jin, Guoqiang Fei, Shaoming Sang, Chunjiu Zhong

**Affiliations:** ^1^Department of Neurology, Zhongshan Hospital, Fudan University, Shanghai, China; ^2^State Key Laboratory of Medical Neurobiology, Collaborative Innovation Center for Brain Science, Fudan University, Shanghai, China; ^3^Shanghai Raising Pharmaceutical Technology Co., Ltd., Shanghai, China

**Keywords:** Alzheimer’s disease, neurodegenerative diseases, bioinformatic analysis, machine learning strategies, biomarkers

## Abstract

**Background:**

Alzheimer’s disease (AD) is the most common neurodegenerative disease, imposing huge mental and economic burdens on patients and society. The specific molecular pathway(s) and biomarker(s) that distinguish AD from other neurodegenerative diseases and reflect the disease progression are still not well studied.

**Methods:**

Four frontal cortical datasets of AD were integrated to conduct differentially expressed genes (DEGs) and functional gene enrichment analyses. The transcriptional changes after the integrated frontal cortical datasets subtracting the cerebellar dataset of AD were further compared with frontal cortical datasets of frontotemporal dementia and Huntingdon’s disease to identify AD-frontal-associated gene expression. Integrated bioinformatic analysis and machine-learning strategies were applied for screening and determining diagnostic biomarkers, which were further validated in another two frontal cortical datasets of AD by receiver operating characteristic (ROC) curves.

**Results:**

Six hundred and twenty-six DEGs were identified as AD frontal associated, including 580 downregulated genes and 46 upregulated genes. The functional enrichment analysis revealed that immune response and oxidative stress were enriched in AD patients. Decorin (DCN) and regulator of G protein signaling 1 (RGS1) were screened as diagnostic biomarkers in distinguishing AD from frontotemporal dementia and Huntingdon’s disease of AD. The diagnostic effects of DCN and RGS1 for AD were further validated in another two datasets of AD: the areas under the curve (AUCs) reached 0.8148 and 0.8262 in GSE33000, and 0.8595 and 0.8675 in GSE44770. There was a better value for AD diagnosis when combining performances of DCN and RGS1 with the AUCs of 0.863 and 0.869. Further, DCN mRNA level was correlated to CDR (Clinical Dementia Rating scale) score (*r* = 0.5066, *p* = 0.0058) and Braak staging (*r* = 0.3348, *p* = 0.0549).

**Conclusion:**

DCN and RGS1 associated with the immune response may be useful biomarkers for diagnosing AD and distinguishing the disease from frontotemporal dementia and Huntingdon’s disease. DCN mRNA level reflects the development of the disease.

## Introduction

Alzheimer’s disease (AD) is the most common neurodegenerative disease that is pathologically characterized by brain β-amyloid (Aβ) deposition forming extracellular plaques, Tau hyperphosphorylation aggregating to intracellular neurofibrillary tangles, and the progressive reduction of synapses and neurons causing brain atrophy (Alzheimer’s Association, 2021). More and more studies also suggest that glial activation underlying neuroinflammation, brain glucose hypometabolism, mitochondrial dysfunction, and oxidative stress also participate in the occurrence and progression of the disease (Liu et al., 2019; Long and Holtzman, 2019). Yet, the pathogenesis of AD remains elusive and the ideal diagnostic biomarkers reflecting the outcome of the disease are still rare.

Genome-wide studies have disclosed the complexity of networks in altered expressions of genes contributing to the pathogenesis of AD. The abnormal modules in immune and microglia-specific activities (Zhang et al., 2013), neural communication, amyloid-β clearance (Magistri et al., 2015), cerebral vasculature (Magistri et al., 2015), and synaptic transmission (Williams et al., 2021) have been indicated the involvement in the disease pathogenesis according to transcriptomics studies on postmortem brain samples of AD patients. The selective regional vulnerability of these involvements and the correlation with clinical outcomes further enhance the complexity (Wang et al., 2016). It leads to significant difficulties in exploring the real pathogenic factor(s) and ideal diagnostic biomarkers for the disease. The recently emerging integrated bioinformatic analysis and machine learning strategy may help to address this challenge. AD, frontotemporal lobar degeneration (FTD), and Huntington’s disease (HD) are all classified into neurodegenerative diseases and they share common neurodegeneration and some clinical symptoms such as cognitive decline (Walker, 2007; Boeve et al., 2022), metabolic changes (Vercruysse et al., 2018; Moll et al., 2020), behavioral and psychological disorders (Santacruz Escudero et al., 2019; Menculini et al., 2021) and so forth, which causes clinically misdiagnosis and mistreatment (Boeve et al., 2022). Accurate diagnostic differentiation also requires expensive PET or aggressive cerebrospinal fluid tests. Meanwhile, these diseases are heterogeneous in pathological mechanisms such as susceptibility of different types of synapses and neuronal cell death (Kamat et al., 2016; Smith-Dijak et al., 2019; John and Reddy, 2021; Wang et al., 2022), which provides the theoretical basis for identifying the differential biomarkers and AD specific pathogenesis. Previous studies have indicated that plasma phosphorylated tau 217 and phosphorylated tau 181 could differentiate AD from FTD (Thijssen et al., 2020, 2021), while a few studies suggested potential biomarkers only on diagnosis of HD without reporting the differential effect from AD (Wild et al., 2008; Battaglia et al., 2011; Caron et al., 2022). Therefore, analyzing the differences in gene expressions of AD, FTD, and HD may provide help for further understanding the pathogenesis and exploring new diagnostic biomarkers of AD differentiating from other neurodegenerative disease.

In this study, we integrated four frontal cortical datasets from the GEO database into an integrated dataset which was used to be compared to one cerebellar dataset of AD, one frontal cortical dataset of FTD, and one frontal cortical dataset of HD, and the results were further verified in another two frontal cortical datasets of AD. We aim at discovering novel pathways and key genes that may serve as valuable diagnostic biomarkers for distinguishing AD from normal patients, FTD, and HD by bioinformatic analysis combined with machine learning strategies. Furthermore, the correlations between the biomarkers with the CDR (Clinical Dementia Rating scale) scores and Braak staging of AD patients were analyzed to explore whether they could also reflect the development of the disease.

## Materials and methods

### Data collection and data processing

The microarray datasets referred to profiles of gene expressions with frontal cortical tissue of brain samples of AD patients were retrieved in National Center for Biotechnology Information (NCBI) Gene Expression Omnibus (GEO) database^1^ with the following keywords including “Alzheimer’s disease,” “Expression profiling by array” and the species was selected as “Homo sapiens.” Four datasets including GSE5281 (10 control and 23 AD samples), GSE131617 (52 control and 19 AD samples), GSE48350 (48 control and 21 AD samples), and GSE84422 (15 control and 24 AD samples) originated from frontal cortical samples with the diagnosis of AD, which are all from the same platform of Affymetrix. Based on the annotation from corresponding datasets, probes were transformed into gene symbols. And if there were more than one probe referring to the same gene symbol, the gene expression values would be assigned as an average value of them. The four AD frontal cortical datasets were normalized with the “SVA” package in R to compensate for the batch effect and merged into an integrated dataset (Leek et al., 2020). The “SVA” package can help remove batch effect in two ways, including estimating surrogate variables for unknown sources of variation and directly removing known batch effects using the “Combat” function (Leek and Storey, 2007, 2008). The integrated AD frontal cortical dataset subtracted the expressed genes in dataset GSE44768 of AD cerebellum to screen frontal-associated genes. Then, the frontal-associated genes of AD were further compared with that in the FTD frontal cortical dataset GSE13162 and HD frontal cortical dataset GSE3790 to identify the candidates of diagnostic biomarkers for AD. More detailed information about the included datasets is summarized in Supplementary Table 1.

### Differential gene expression analysis

Differentially expressed genes (DEGs) were analyzed with the “limma” package in R and identified with the thresholds that | log2FC| (fold change) was larger than 2 and the adjusted *p*-value was less than 0.05. After subtracting the DEGs of the AD cerebellar dataset and comparing them with that in FTD and HD frontal cortical datasets, DEGs only expressed in AD frontal cortex were identified. Besides, heatmaps and volcano plots were conducted with “pheatmap” and “EnhancedVolcano” packages in R. Venn diagrams were used to visualize the overlapping or unique genes among AD cortex, AD cerebellum, HD, and FTD (Bardou et al., 2014).

### Functional enrichment analysis

Gene ontology (GO) enrichment analysis was performed under three hierarchical categories of biological process, molecular function, and cellular component with the “clusterProfiler” package in R (Yu et al., 2012). Kyoto Encyclopedia of Genes and Genomes (KEGG) database was applied to do the pathway enrichment analysis with the “clusterProfiler” package in R (Yu et al., 2012; Yu and He, 2016; Minoru et al., 2017; Jassal et al., 2020).

### Machine learning for potential diagnostic biomarkers

We employed three machine learning strategies to identify potential diagnostic biomarkers differentiating Alzheimer’s disease from other neurodegenerative diseases. Least absolute shrinkage and selection operator (LASSO) logistic regression was applied to identify the diagnostic genes associated with discrimination with the “glmnet” package in R (Tibshirani, 1996; Bühlmann, 2011). Support vector machine-recursive feature elimination (SVM-RFE) was conducted in R using the “e1071” package with 5-fold cross-validation (Suykens and Vandewalle, 1999; Weston and Guyon, 2012). Random forest (RF) was performed in R with the “randomForest” package (Breiman, 2001; Qi, 2012). Then, the overlapping genes of the above three machine-learning strategies were identified and would be further verified as candidate biomarkers.

### Validation and evaluation of candidate biomarkers

Two additional AD frontal cortical datasets (GSE33000 and GSE44772) were applied as the validation datasets. The overlapping genes with differential expression identified in the above three mentioned machine-learning strategies were validated. The receiver operating characteristic curves (ROCs) analysis was conducted and the area under the curves (AUCs) was calculated to evaluate the diagnostic efficacy of the selected biomarkers (Seshan et al., 2013). Besides, CDR scale is an informant-based global clinical instrument which has been widely applied for clinical grading of dementia severity (Schafer et al., 2004; Huang H. et al., 2021). Based on the neurofibrillary tangle topographic distribution in the brain, the Braak staging is strongly associated with the degree of cognitive impairment (Braak and Braak, 1991; Nelson et al., 2012; Lowe et al., 2018). Thus, the correlation analysis between the diagnostic biomarkers and the CDR scores and Braak staging was also performed in dataset GSE84422 to explore whether they could reflect the development of the disease.

## Results

### Identification of differentially expressed genes in the frontal cortex of AD

**FIGURE 1:**
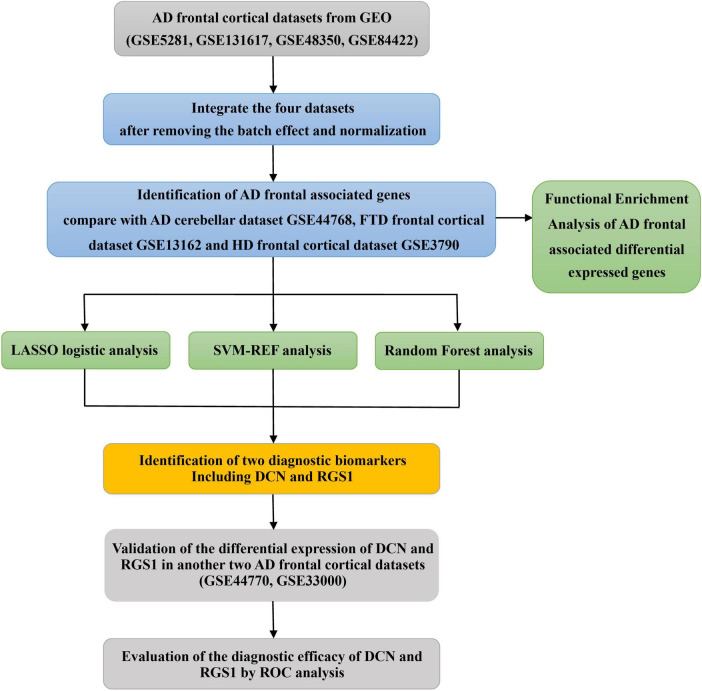
The workflow of the analysis process.

A diagram of the workflow of the bioinformatics analyses combined with machine learning strategies is shown in Figure 1.

**FIGURE 2:**
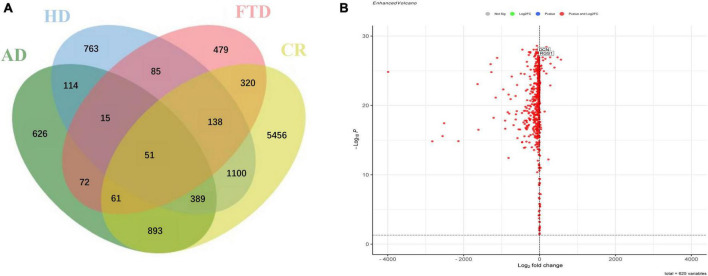
Identification of specific differentially expressed genes in the frontal cortex of Alzheimer’s disease. **(A)** Venn diagram showed the intersection of differentially expressed genes in frontal cortical samples of Alzheimer’s disease (AD), cerebellar samples of Alzheimer’s disease (CR), frontal cortical samples of frontotemporal lobar degeneration (FTD), and frontal cortical samples of Huntington’s disease (HD). **(B)** Volcano plot of frontal specific differentially expressed genes between Alzheimer’s disease and controls.

After removing batch effects, the data of expression profiles of frontal cortical samples of AD from four GEO datasets (GSE5281, GSE131617, GSE48350, and GSE84422) were merged into an integrated dataset, including frontal cortical samples of 87 AD patients and 125 control subjects. Firstly, the DEGs in the frontal cortical samples of AD from the integrated dataset, cerebellar samples of AD (GSE44768), frontal samples of FTD (GSE13162), and HD (GSE3790) patients were all analyzed. Secondly, the frontal-associated genes of AD were screened, and selected the DEGs in the frontal cortex of AD patients as compared with control subjects, while not in the cerebellum of AD patients as compared with control subjects. Then, the frontal-associated genes were further compared with those in FTD and HD datasets to finally identify the candidates of diagnostic biomarkers for AD. The results showed that 626 DEGs were AD-associated, including 580 downregulated genes and 46 upregulated genes. The Venn diagram showed the intersection of the DEGs from the above-mentioned datasets (Figure 2A). The volcano plot described AD frontal associated gene distribution (Figure 2B), and the heatmap illustrated the remarkable differences (Supplementary Figure 1).

### Functional enrichment analyses

**FIGURE 3:**
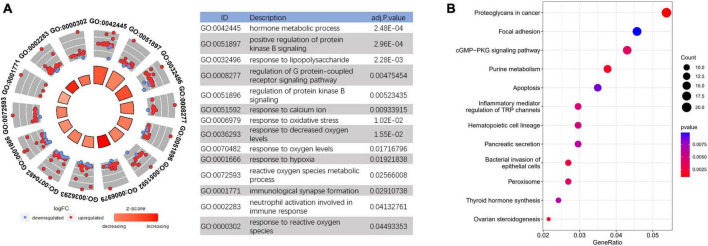
Functional enrichment analysis to investigate the potential function of differentially expressed genes (DEGs). **(A)** GO analyses of DEGs. **(B)** Kyoto Encyclopedia of Genes and Genomes (KEGG) pathway analysis of DEGs.

Based on the DEGs in AD frontal cortex, GO analysis was performed to explore the main cell functions, most of which were associated with the immune response and oxidative stress-related such as response to lipopolysaccharide, neutrophil activation, response to oxidative stress, reactive oxygen species metabolic process, etc. (Figure 3A and Supplementary Table 1). KEGG pathway analysis was also conducted and suggests significant enrichment in pathways of the cGMP-PKG signaling pathway, Peroxisome, Ras signaling pathway, etc. (Figure 3B).

### Screening and verification of the diagnostic markers

**FIGURE 4:**
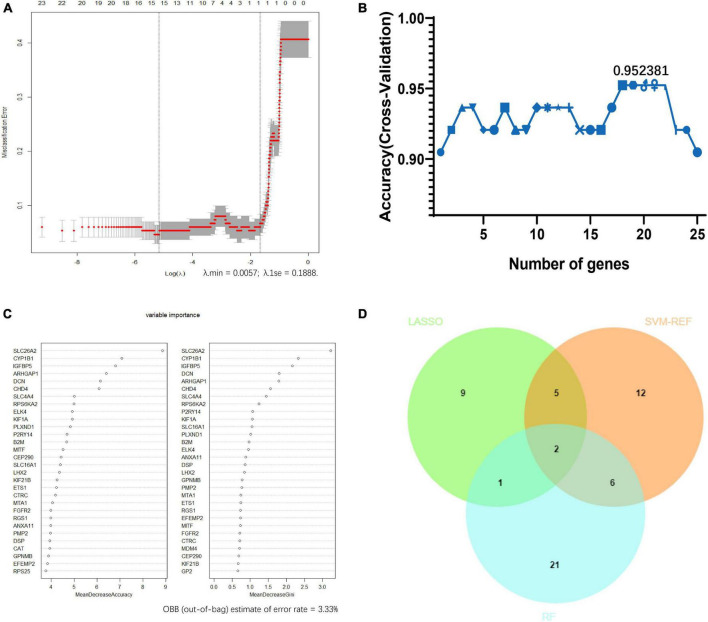
Screening candidate biomarkers of Alzheimer’s disease via machine-learning strategies. **(A)** Least absolute shrinkage and selection operator (LASSO) logistic regression. **(B)** Support vector machine-recursive feature elimination (SVM-RFE) analysis. **(C)** Random forest (RF) analysis. **(D)** Venn diagram showed the intersection of diagnostic markers obtained by the three machine learning strategies.

Least absolute shrinkage and selection operator logistic regression algorithm identified 17 potential diagnostic markers from AD frontal-associated DEGs (Figure 4A). SVM-RFE and RF analysis showed there were 25 and 30 potential diagnostic markers for AD, respectively (Figures 4B, C). Among these potential biomarkers, there were two overlapping genes including decorin (DCN), and regulator of G protein signaling 1 (RGS1) (Figure 4D).

**FIGURE 5:**
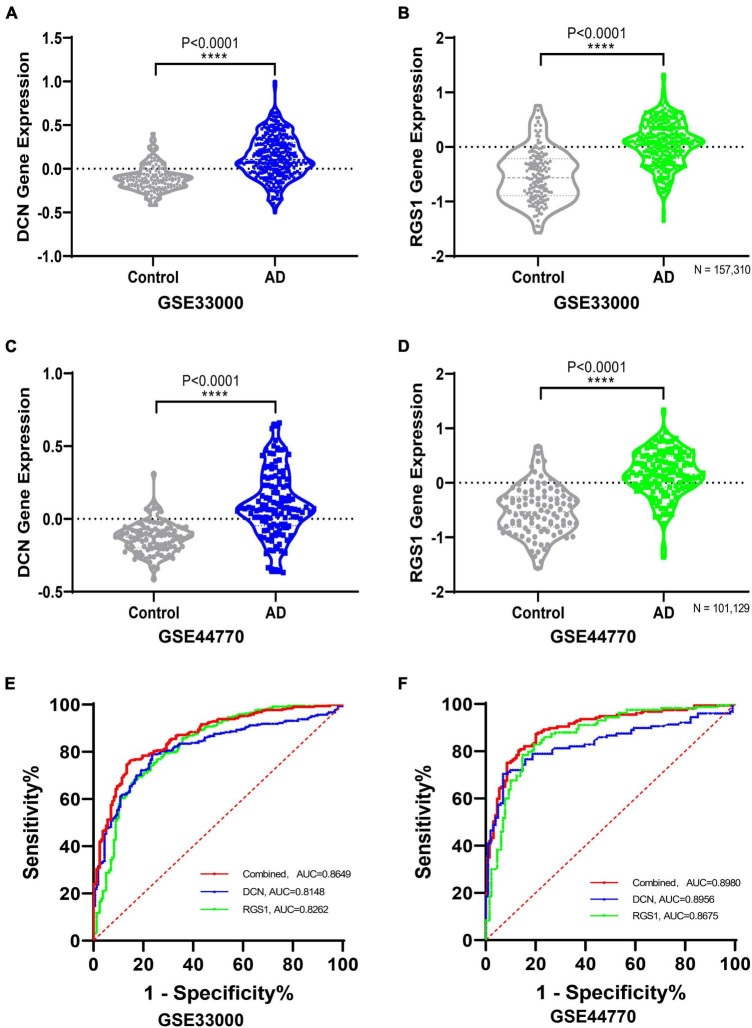
Validating the differential expressions of diagnostic biomarkers in verification datasets. **(A)** Validation of the expression levels and diagnostic efficacy of DCN in dataset GSE33000. **(B)** Validation of the expression levels of RGS1 in dataset GSE33000. **(C)** Validation of the expression levels of DCN in dataset GSE44770. **(D)** Validation of the expression levels of RGS1 in dataset GSE44770. **(E)** ROC analysis of DCN, RGS1, and the combination of the two biomarkers as a diagnostic tool in the validation dataset GSE33000. **(F)** Receiver operating characteristic (ROC) analysis of DCN, RGS1, and the combination of the two biomarkers as a diagnostic tool in the validation dataset GSE44770.

**FIGURE 6:**
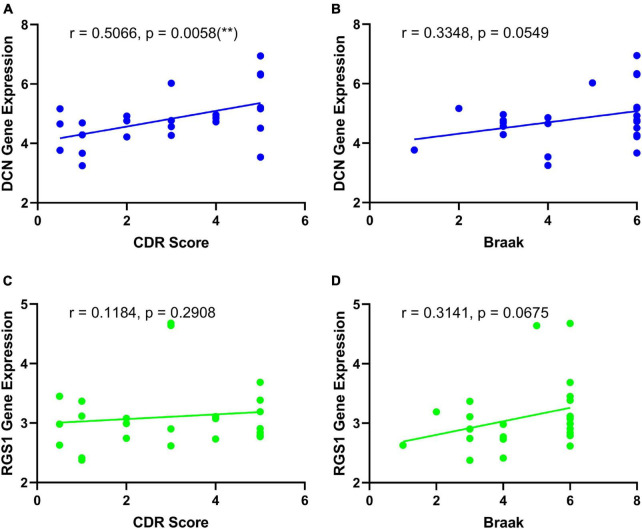
Correlation analysis of the diagnostic biomarkers and the development of disease. **(A)** Correlation analysis of DCN and CDR score. **(B)** Correlation analysis of DCN and Braak staging. **(C)** Correlation analysis of RGS1 and CDR score. **(D)** Correlation analysis of RGS1 and Braak staging.

The differential expressions of DCN and RGS1 were further validated in two additional datasets GSE33000 and GSE44770, and the results suggested that both of them were significantly upregulated (Figure 5 and Supplementary Table 1). The ROC analysis of DCN and RGS1 was conducted to assess the diagnostic efficacy. The AUCs of DCN and RGS1 were 0.8148 and 0.8262 in dataset GSE33000, and 0.8595 and 0.8675 in GSE44770 (Figure 5E). The AUCs of the combination of the two biomarkers as a diagnostic tool for AD were 0.863 and 0.869 (Figure 5F). DCN mRNA level was correlated to CDR (Clinical Dementia Rating scale) score (*r* = 0.5066, *p* = 0.0058) and Braak staging (*r* = 0.3348, *p* = 0.0549) (Figures 6A, B). RGS1 mRNA level correlates with Braak staging (*r* = 0.3141 *p* = 0.0675) and CDR score (*r* = 0.1184, *p* = 0.2908) (Figures 6C, D).

## Discussion

Cerebellum has always been viewed as a less affected or even neglected area of AD because autopsy studies found that the two main neuropathological hallmarks of AD including amyloid plaques and neurofibrillary tangles are both present first in the cerebral cortex while the cerebellum could escape from neurofibrillary tangles (Larner, 1997). Although the emerging role of the cerebellum in AD has gained some attention, recent studies revealed that the genomic changes mainly occurred in the cortex instead of the cerebellum, especially excitatory neurons are preferentially vulnerable to pathological changes in AD (Fu et al., 2019; Leng et al., 2021; Miller et al., 2022). Multiple cerebral regions have been reported to be related to AD (Mu and Gage, 2011; Aggleton et al., 2016; Cogswell et al., 2021), among which the frontal cortex is more predictive of cognitive impairments and early disturbance of daily activities, which gives rise to more sensitivity to subclinical changes (Stoeckel et al., 2013; Marshall et al., 2019). Therefore, to identify biomarkers that are more related to the pathogenesis and clinical manifestations of AD and avoid interference from non-AD-associated genes, we selected DEGs in the frontal cortex of AD patients as compared with control subjects, while not in cerebellum AD patients as compared with control subjects. Those DEGs were defined as AD-associated genes.

In the current studies, in order to solve the problem of the small sample size of existing individual databases originating from frontal samples of AD patients, we first integrated four homogeneous databases into an integrated database with a larger sample size. Then, the frontal-associated DEGs of AD were screened by comparing the differences in the DEGs between the integrated and cerebellar datasets (Figure 2A). AD, HD, and FTD are neurodegenerative diseases with cognitive impairment, which clinically need differential diagnosis. To further identify the potential biomarkers for AD diagnosis, the frontal-associated DEGs and the DEGs from the datasets of FTD and HD frontal samples were analyzed. The 626 AD-associated DEGs were identified, including 580 upregulated genes and 46 downregulated genes (Figure 2B). The functional enrichment analysis revealed that the immune response and oxidative stress-related processes were highly enriched such as neutrophil activation, immunological synapse formation, response to oxidative stress, reactive oxygen species, and so forth (Figure 3). As mounting genetic and functional evidence indicates, neuroinflammation is a prominent manifestation of AD, and the immune response does matter a lot in the pathogenesis of the disease (Gjoneska et al., 2015; Shi and Holtzman, 2018). The immune response in AD is mobilized by the immune components in the central nervous system (CNS) mainly including complement and microglia, which function as a “double-edged sword.” Complement proteins not only act as the first line of defense to help synaptic pruning, mediate Aβ clearance and maintain neural circuits (Schafer et al., 2012; Hong et al., 2016) but associate with Aβ plaques causing the surrounding neuronal atrophy and synapse loss (Hong et al., 2016; Jevtic et al., 2017; Shi et al., 2017). As one of the prominent immune cells of the CNS, microglia rapidly respond to stimuli and pathogens performing immune surveillance, phagocytosis, and neuroprotection (Streit et al., 1999; Davalos et al., 2005; Nimmerjahn et al., 2005). However, the inflammatory cytokines and oxidative stress produced by microglia also lead to neural impairment (Heneka et al., 2013; McIntosh et al., 2019; Marschallinger et al., 2020; Hashioka et al., 2021). Recent studies also suggest that oxidative stress causes neuronal damage in various pathways, and plays a critical or even center role pathogenesis and mechanism of AD (Jiang et al., 2016; Bai et al., 2022). Oxidative stress promotes the expression of amyloid precursor protein (APP) and upregulates the activity of β-secretase contributing to the aggregation of Aβ (Tamagno et al., 2012; Butterfield and Boyd-Kimball, 2018). Oxidative stress can also enhance the Tau phosphorylation by producing reactive oxygen species (ROS) and directly interacting with glycogen synthase kinase-3 (GSK3) (Su et al., 2010; Du et al., 2022).

Most importantly, DCN and RGS1 were identified to be useful diagnostic biomarkers by integrated bioinformatic analysis and machine-learning strategies. The diagnostic efficacies of DCN and RGS1 were verified by ROC analysis with the AUCs of 0.8148 and 0.8262 in dataset GSE33000, and 0.8595 and 0.8675 in GSE44770. And the combination of the two biomarkers as a diagnostic tool for AD owned even higher AUCs of 0.863 and 0.869 in the validated datasets. Furthermore, the correlation analysis indicated that DCN was significantly correlated to the CDR score and may be correlated to the Braak stage, and the RGS1 showed a significant correlation to the Braak stage. The CDR is one of the most widely used global clinical rating scales to evaluate the development of disease (Morris, 1993). Braak staging is based on the neurofibrillary tangle topographic distribution in the brain and is strongly associated with cognitive impairment (Braak and Braak, 1991). Therefore, DCN and RGS1 may reflect the development of the disease.

Decorin is an extracellular matrix proteoglycan associated with collagen fibril formation (Reed and Iozzo, 2002). Recent researches have shown that it also plays a critical role in autoimmune and inflammatory diseases (Dong et al., 2022), antifibrotic (Järvinen and Ruoslahti, 2019), antioxidant, and antiangiogenic properties (Sofeu Feugaing et al., 2013; Järveläinen et al., 2015). DCN was found significantly increased in both AD mouse models and CSF of AD patients, predicting well innate immune activation and potential choroid plexus dysfunction (Jiang et al., 2022). Other studies also demonstrated that endothelial-activated DCN-positive astrocytes contributed to vascular amyloid deposits but not parenchymal amyloid plaques in AD mouse models and AD/CAA patients (Taylor et al., 2022). Our study here confirmed that DCN was significantly increased in AD brain parenchyma at the mRNA level and could perform as a useful diagnostic marker. Future study is still needed to clarify the source and function of AD pathogenesis.

RGS1 is one of the members of the RGS family and is identified as an immediate early gene responding to B cell activation signals (Liang et al., 2018). RGS1 serves as a negative regulator to inhibit the chemotaxis of cytotoxic T lymphocytes and TH1 cells toward tumor-associated chemokines (Huang D. et al., 2021). It was also found to regulate the homeostasis and trafficking of B and T cells to inflammatory diseases and attract macrophages to atherosclerotic plaques (Patel et al., 2015). At present, research on the association between RGS1 and AD is limited. Only one study has shown that RGS1 was upregulated in the PBMCs of AD patients (Leandro et al., 2018). Therefore, RGS1 is expected to be a blood-based diagnostic marker, and the role of RGS1 in AD is worthy of further study.

There are some limitations to our study. First, although we carefully select the same frontal lobe of brain samples of AD from the same platforms of Affymetrix and have validated them in another two datasets, the results still need to be thoroughly confirmed experimentally. Second, limited by the sample size, the availability of the biomarkers in differentiating from other neurodegenerative diseases and reflecting the development of the disease needs further clinical exploration. Third, lacking appropriate datasets of the frontal cortex with a diagnosis of HD and FTD from the same platform as the selected datasets of AD, we failed to further evaluate the differential effect of biomarkers distinguishing AD from HD and FTD. We would like to update this evaluation, once there were appropriate datasets.

## Conclusion

We applied an approach of integrated bioinformatic analysis combined with machine learning strategies to identify AD frontal-associated biomarkers differentiating from normal subjects, FTD and HD. The results revealed that DCN and RGS1 associated with immune response are useful biomarkers for diagnosing AD and distinguishing from FTD and HD, and their mRNA levels may also reflect the development of the disease.

## Data availability statement

The original contributions presented in this study are included in the article/Supplementary material, further inquiries can be directed to the corresponding authors.

## Author contributions

SS and CZ designed the study and accessed the funding. BJ downloaded the data and perform bioinformatics analysis. SS, GF, and BJ wrote the manuscript. All authors contributed to the article and approved the submitted manuscript.

## Footnotes

1
https://www.ncbi.nlm.nih.gov/geo/
