# Supplementary figures and images for: Identification of biomarkers differentiating Alzheimer’s disease from other neurodegenerative diseases by integrated bioinformatic analysis and machine-learning strategies
Source: Front Mol Neurosci. 2023 May 10;16:1152279. doi: 10.3389/fnmol.2023.1152279 (PMC10205980; doi:10.3389/fnmol.2023.1152279)

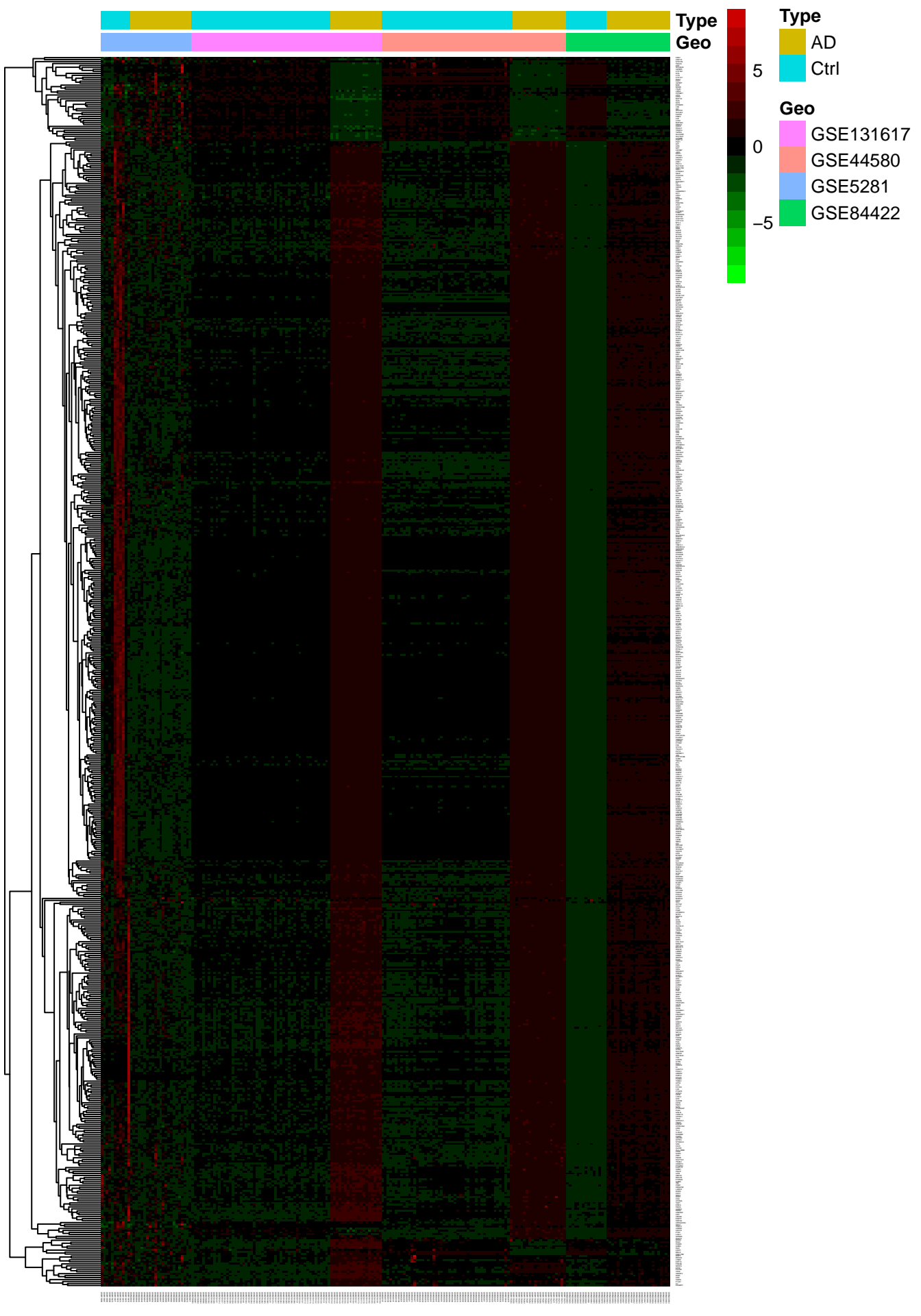

Supplement: Supplementary file 1 [file Data_Sheet_1.PDF]
